# Amylases and Their Importance during Glycan Degradation: Genome Sequence Release of *Salmonella* Amylase Knockout Strains

**DOI:** 10.1128/genomeA.00355-17

**Published:** 2017-05-18

**Authors:** Narine Arabyan, Bihua C. Huang, Bart C. Weimer

**Affiliations:** a Department of Population Health and Reproduction, School of Veterinary Medicine, University of California, Davis, Davis, California, USA; b 100K Pathogen Genome Project, UC Davis, Davis, California, USA

## Abstract

Amylases catalyze the cleavage of α-d-1,4 and α-d-1,6-glycosidic bonds in starch and related carbohydrates. Amylases are widely distributed in nature and are important in carbohydrate metabolism. This is the release of four single and two double deletions in *Salmonella enterica* serovar Typhimurium LT2 that are important for glycan degradation during infection.

## GENOME ANNOUNCEMENT

Amylases in *Salmonella* spp. are poorly characterized glycosyl hydrolases. The importance of amylases during *Salmonella* infection is understudied, especially as new virulence factors. The genome of *Salmonella enterica* serovar Typhimurium LT2 contains four amylases that are classified under the glycoside hydrolase 13 (GH13) family: *malS* (periplasmic α-amylase), *amyA* (cytoplasmic α-amylase), *glgX* (glycogen-debranching enzyme), and *glgB* (1,4-α-glucan-branching enzyme). Although *glgX* and *glgB* are not annotated as amylases, both have a conserved α-amylase domain and function as amylases. Investigating the genomic diversity of microbial amylases will aid in understanding their importance during pathogenesis. Inhibitors of amylases can be new drug targets.

Amylases play an important role by altering the host glycan profiles during infection to gain access of the host epithelial cells by binding to terminal mannose molecules to initiate glycan degradation, as was shown by Arabyan et al. (1). That group also demonstrated that each of the four mutant amylase strains (Δ*malS,* Δ*amyA,* Δ*glgX*, and Δ*glgB* mutants) had different invasion phenotypes during the *in vitro* infection of differentiated colonic epithelial cells (Caco-2) (1); however, only the Δ*malS* mutant significantly (*P* < 0.05) reduced adhesion and invasion during infection that were comparable to those seen with a nonpathogenic *Salmonella* strain (1).

The 100K Pathogen Genome Project (http://www.100kgenomes.org) is a large-scale sequencing consortium that offers the use of new next-generation sequencing methods to provide cutting-edge methods for pathogen detection and control in the food supply. This project is focused on producing genomes of pathogenic isolates from the environment, plants, animals, and humans worldwide, providing new insights into the genetic diversity of *Salmonella* and other foodborne pathogens. These amylase mutant strains (four single-deletion and two double-deletion mutants) were constructed in the Weimer laboratory (UC Davis, Davis, CA) (1), as described by Datsenko and Wanner (2). Cultures were grown on 1.5% Luria-Bertani (LB) agar (Difco, Franklin Lakes, NJ) with 10 µg/ml chloramphenicol at 37°C and lysed (3); genomic DNA (gDNA) was extracted (4), checked for quality (5), and fragmented (6). Libraries were 350 to 500 bp (7, 8) and were indexed (96 genomes/lane) and sequenced (Illumina HiSeq 3000; PE150) (9–11) at UC Davis DNA Technologies Core (Davis, CA). Paired-end reads were *de novo* assembled using CLC Workbench version 6 (Qiagen), with default parameters. Here, the 100K Pathogen Genome Project has assembled four genomes of single- and two double-amylase-deletion strains of *Salmonella enterica* serovar Typhimurium LT2.

### Accession number(s).

All sequences are publicly available and can be found at the 100K Project BioProject (NCBI PRJNA186441) in the Sequence Read Archive (http://www.ncbi.nlm.nih.gov/sra) and genome assemblies can be found in NCBI GenBank (Table 1).

**TABLE 1:** *Salmonella enterica* serovar Typhimurium LT2 amylase deletion mutants

GenBank accession no.	SRA accession no.	Isolate name	Mutation	No. of contigs	Coverage (×)	Total genome size (bp)	No. of CDSs^a^
MXAY00000000	SRR3622951	BCW_7501	Δ*malS*	65	105	4,892,783	4,797
MZNJ00000000	SRR5288769	BCW_7502	Δ*glgB*	64	287	4,896,277	4,809
MXAZ00000000	SRR3622952	BCW_7503	Δ*glgX*	50	130	4,893,261	4,799
MZNK00000000	SRR5288768	BCW_7513	Δ*invA*::Δ*malS*	61	150	4,893,696	4,809
MZNL00000000	SRR5288767	BCW_7521	Δ*flgJ*::Δ*malS*	57	139	4,896,778	4,803
MZNM00000000	SRR5288770	BCW_8419	Δ*amyA*	58	150	4,892,055	4,802

a CDSs, coding sequences.
